# Prognostic model for nephrotoxicity among HIV-positive Zambian adults receiving tenofovir disoproxil fumarate-based antiretroviral therapy

**DOI:** 10.1371/journal.pone.0252768

**Published:** 2021-07-12

**Authors:** Freeman W. Chabala, Edward D. Siew, Wilbroad Mutale, Lloyd Mulenga, Aggrey Mweemba, Fastone Goma, Njeleka Banda, Patrick Kaonga, William C. Wester, Douglas C. Heimburger, Muktar H. Aliyu, Derick Munkombwe

**Affiliations:** 1 Levy Mwanawasa Medical University, Institute of Basic and Biomedical Sciences, Lusaka, Zambia; 2 The University of Zambia, School of Medicine, Lusaka, Zambia; 3 Vanderbilt University Medical Center, Division of Nephrology and Hypertension, Vanderbilt O’Brien Center for Kidney Disease, Nashville, Tennessee, United States of America; 4 Tennessee Valley Health Systems (TVHS), Veterans Affairs, Nashville, TN, United States of America; 5 The University of Zambia, School of Public Health, Lusaka, Zambia; 6 Vanderbilt Institute for Global Health, Vanderbilt University Medical Center, Nashville, TN, United States of America; 7 The University Teaching Hospital, Lusaka, Zambia; 8 Meharry Medical College School of Medicine, Nashville, TN, United States of America; 9 The University of Zambia, School of Health Sciences, Lusaka, Zambia; Johns Hopkins University, UNITED STATES

## Abstract

Persons living with HIV (PLWH) receiving tenofovir disoproxil fumarate (TDF)-based antiretroviral therapy (ART) risk suffering TDF-associated nephrotoxicity (TDFAN). TDFAN can result in short- and long-term morbidity, including permanent loss of kidney function, chronic kidney disease (CKD), and end-stage kidney disease (ESKD) requiring dialysis. Currently, there is no model to predict this risk or discern which patients to initiate TDF-based therapy. Consequently, some patients suffer TDFAN within the first few months of initiating therapy before switching to another suitable antiretroviral or a lower dose of TDF. In a prospective observational cohort study of adult Zambian PLWH, we modelled the risk for TDFAN before initiating therapy to identify individuals at high risk for experiencing AKI after initiating TDF-based therapy. We enrolled 205 HIV-positive, ART-naïve adults initiating TDF-based therapy followed for a median of 3.4 months for TDFAN at the Adult Infectious Disease Research Centre (AIDC) in Lusaka, Zambia. We defined TDFAN as meeting any of these acute kidney disease (AKD) criteria: 1) An episode of estimated glomerular filtration rate (eGFR)< 60ml/ min/1.73m^2^ within 3 months, 2) reduced eGFR by> 35% within 3 months or 3) increased serum creatinine by> 50% within 3 months. A total of 45 participants (22%) developed acute kidney disease (AKD) after TDF-based therapy. The development of AKD within the first 3 months of commencing TDF-based therapy was associated with an increase in baseline serum creatinine, age, baseline eGFR and female sex. We concluded that baseline characteristics and baseline renal function biomarkers predicted the risk for AKD within the first 3-months of TDF-based therapy.

## Introduction

Human Immunodeficiency Virus (HIV) infection remains a major global public health problem, with 38 million people currently living with HIV and close to 700,000 AIDS-related deaths recorded in 2019 [1]. This burden is most profound in sub-Saharan Africa, where more than two-thirds (70%) of persons living with HIV (PLWH) currently reside and more than half (52%) of all HIV-related deaths take place [1]. Zambia, a low-to-middle income country (LMIC) with a population of 18.4 million is responsible for approximately 21% of Africa’s HIV disease burden and almost 5% of AIDS-related deaths on the continent [1]. Whereas the widespread availability of combination antiretroviral therapy (ART) has dramatically attenuated the impact of HIV disease [2], ART is not without potential short- and longer-term adverse effects [2–4].

A common first-line antiretroviral (ARV) medication tenofovir disoproxil fumarate (TDF) causes nephrotoxicity that can damage proximal tubules and manifest as acute kidney injury (AKI) [5–9]. The latter is associated with an increased risk of death and morbidity [10–13], including chronic kidney disease (CKD), end-stage kidney disease (ESKD) and other indirect complications, including cardiovascular diseases (CVD) [14–17].

The incidence of AKI in HIV ranges between 5% to 22%, depending on the study site and modality of outcome selected [18–20]. Despite some studies asserting that AKI in HIV is associated with many factors [21–23], others contend that TDF is the main perpetrator [13, 24, 25]. Predominantly, TDF-associated nephrotoxicity occurs in the first three to six months of initiating therapy [26–28]. Unfortunately, patients in Zambia are treated empirically without estimating the risk for TDF-associated nephrotoxicity because to our knowledge there are no uniformly accepted risk models available to predict which patients are at heightened risk for adverse kidney outcomes prior to initiating ART. Consequently, ART-treated adults experiencing TDF-associated nephrotoxicity are either switched to an abacavir (ABC)-based regimen or placed on a lower dose of TDF. However, even after these ART regimen modifications, some studies argue that following an episode of AKI the patients remain at high risk for its complications [12, 29]. Therefore, this study was designed to derive and internally validate a predictive model for TDF-associated nephrotoxicity before initiating therapy to help physicians effectively discriminate patients to treat with TDF from those for whom it should be avoided to prevent iatrogenic nephrotoxicity.

## Methods

### Study design

This was a prospective observational cohort study of 205 participants systematically randomly sampled to include every other ART-naïve adult that the clinician initiated on TDF-based therapy to participate in the study. The study was done within the confines of standard clinical practice, where only candidates that the clinicians initiated on TDF-based therapy were invited to participate in the study. The investigators had no control over whom the clinicians initiated on therapy; they initiated participants based on the Zambian national ART treatment guidelines [30] and their expert clinical discretion of the risk for TDF-associated nephrotoxicity.

We prospectively observed the participants for approximately three months to project the risk of TDF-associated nephrotoxicity in the first 3 months of therapy [26–28]. Kidney function was assessed according to Zambian national HIV treatment guidelines [30] on the day of beginning TDF-based therapy (baseline) and after 3 months of therapy. The primary outcome variable was the presence of TDF-associated nephrotoxicity at whichever visit date was closest to three months of TDF-based therapy initiation. However, approximately 15% of participants did not report for kidney function assessments at exactly three months from baseline; we, therefore, accepted kidney function assessment results obtained ± 1 month from 3-months which lead to no missing data on outcomes. We considered participants lost to follow-up if we were unable to reach them through the contacts they provided or if no subsequent kidney function results for the outcome visit were traceable in the Laboratory Information System (LIS) other than the information we collected on the day of enrolment.

The outcome variable (TDF-associated nephrotoxicity) was defined as the presence of acute kidney disease (AKD) [31] at the 3-month visit; participant having either: 1) An episode of eGFR < 60ml/ min/1.73m^2^ within 3 months, 2) reduction in eGFR by greater than 35% within 3 months, or 3) increase in serum creatinine by more than 50% within 3 months [32, 33].

### Study site and sources of data

Permission to conduct the study was granted by the management of University Teaching Hospital, Lusaka. The hospital is Zambia’s largest ART treatment and referral centre. The data were collected from participants that visited the Adult Infectious Disease Centre, the national HIV referral centre for care and follow-up of PLWH located within the hospital premises. The research nurse identified participants that clinicians initiated on a TDF-based regimen and invited them to participate in the study. Participants were given information sheets, any questions related to the study were answered, and those that voluntarily accepted to participate signed an informed consent form. The study nurse interviewed participants and collected demographics and health history using REDCap; further, she validated and obtained additional information (other treatment and comorbidities) from the patient’s paper files, and SmartCare, an electronic health record (EHR) system. SmartCare is a national HIV EHR system deployed by the Zambian Ministry of Health in collaboration with the U.S. Centers for Disease Control and Prevention (CDC).

### Participant recruitment and eligibility

The University of Zambia Biomedical Research Committee (UNZABREC) granted ethical approval and National Health Research Authority (NHRA) permitted the study. Participants were recruited and followed up from 24^th^ of December 2018 to 16^th^ January 2020. The clinicians initiated participants on therapy according to the Zambian national ART guidelines [30] that recommend initiating TDF-based treatment in patients with an intact/preserved kidney function (estimated glomerular filtration rate (eGFR)≥ 60 mL/min/1.73m^2^ estimated using the CKD-EPI formula and no known history of kidney disease). In their assessment for an intact/preserved kidney function and minimal risk for TDF-associated nephrotoxicity, some clinicians checked for a history of cardiovascular diseases (CVDs), but this was not standard practice and not performed routinely. The CVDs were defined as any diseases of the heart and blood vessels and included congenital heart diseases, rheumatic heart diseases, coronary heart diseases, and cerebral vascular diseases We excluded candidates previously on other ART regimens, and vulnerable candidates who could not effectively decline participation if not willing, and prisoners.

A total of 452 eligible candidates were identified, but approximately one-half were not selected due to systematic random selection of the second candidate; another 18 selected candidates were excluded because they had a history of being on ART elsewhere. Three candidates were excluded because they were prisoners, which left a total of 205 outpatient adult males and females (≥18 years of age) for observation (S1 Fig). Of the 205 enrolled participants, two participants were excluded from follow up but were included in the analysis because they declined to provide blood and urine samples for laboratory analysis at baseline and subsequent visits, but did provide their demographic information. Therefore, 205 participants were included in the analysis because we imputed the kidney outcomes for the two.

The study nurse approached the participants immediately after being prescribed TDF-based therapy by the clinician and interviewed them to establish any history of comorbidities, such as diabetes mellitus, chronic diarrhoea, hepatitis B, hepatitis C or tuberculosis, concomitant traditional medication use, non-steroidal autoinflammatory drugs use, and lifestyle choices, such as smoking and alcohol use. None of the participants self-reported or had documented history of the above comorbidities.

### Biosample measures

We collected blood and urine samples on the day of TDF-based therapy initiation (enrollment and baseline), and at three months of TDF-based therapy. Approximately 4 mL of blood samples were collected in an EDTA vacutainer, potassium oxalate, lithium heparinized vacutainer and plain vacutainer. We measured variables related to kidney function in both blood and urine samples collected at baseline as the predictor and measured kidney outcomes from the 3-month samples. Some of the laboratory tests performed relevant to this study included viral load, CD4+ count, serum/urine creatinine, serum sodium, serum potassium, serum/urine phosphate, serum cholesterol, serum haemoglobin, blood urea nitrogen, serum glucose, and urine protein. Viral suppression was defined as viral load < 50copies/mL) and viremia was defined as viral load> 1000 copies/mL. The Bechman Coulter AU480 chemistry analyzer was used to analyze all clinical chemistry samples (Bechman Coulter, Midrand, South Africa). CD4+ cell count was measured using the Becton Dickinson (BD) FACS Calibur (BD Biosciences, Erembodegem, Belgium), and viral load using COBAS® Ampliprep/ COBAS® Taqman 48 HIV-1 Tests version 2 (Roche Diagnostics Corporation, Indiana, USA) and the Hologic Panther (Hologic, Massachusetts, USA). Finally, haemoglobin was analyzed using the Sysmex XT 4000i haematology analyzer (Sysmex Corporation, Dubai, UAE).

### Sample size justification

The sample size was estimated using coefficients obtained from analyzing Nutritional Support for African Adults Starting Antiretroviral Therapy (NUSTART)-nested study [34]. A random pilot sample of 50 adult male and females treated with TDF-based therapy from baseline to 3-months was analyzed to estimate the hazard ratio of TDF-associated nephrotoxicity. We studied 1 female per male subject accrued for 12 months with an additional follow-up of 3 months after accrual. The median survival time amongst females was 2.69 months. If the true hazard ratio of females relative to males was 1.68, we needed to study 75 males and 75 females initiating TDF-based therapy to be able to reject the null hypothesis that the TDF-associated nephrotoxicity survival curves of male and females were equal with 80% power and type I error probability of 0.05. Assuming a loss to follow-up of 10%, we needed to enrol 185 participants.

### Data management and analysis

Study data were collected and managed using REDCap electronic data capture tools (https://projectredcap.org/) and exported into Stata 15 (StataCorp LCC., College Station, Texas, USA) for analysis [34, 35]. Categorical variables were summarized using proportions. The difference in proportions of binary variables was computed using Pearson’s chi-square test or Fischer’s exact test. The normality of distribution was tested using the Shapiro-Wilk test. Median and interquartile ranges summarized continuous variables. The difference between the two medians was tested by Wilcoxon’s Mann-Whitney rank-sum test and Student’s *t-*test for actual mean differences. The baseline covariates that were considered important predictors of AKD include: patient age, sex, BMI, urine albumin-to-creatinine ratio, CD4+ count, mean arterial pressure (MAP), viral load, serum creatinine and eGFR. Data reduction was by subject knowledge from the literature search, and hierarchical variable clustering (S2 Fig) to eliminate collinearity and inconsistent variables. Hierarchical variable clustering eliminated ordinal eGFR categorized (KDIGO); and ordinal proteinuria; while Spearman index (S3 Fig) selected age, sex, BMI, baseline eGFR, baseline serum creatinine, mean arterial pressure, CD4+ count, log-transformed viral load, and log-transformed urine albumin-to-creatinine ratio into the final model. The Cox regression model determined the association between the baseline predictors and TDF-associated nephrotoxicity at 3-months. Proportional hazards assumption was tested using Log-log plots and unrestricted Kaplan-Meier plots (S4 and S5 Figs) and multicollinearity were tested using the variance-covariance matrix estimators (S1 Table). Harrell’s concordance and Somers’ D determined model discrimination. We used internal model validation by Efron’s bootstrap and corrected Harrell’s concordance, and Somers’ D for calibration error (heuristic shrinkage error estimator) [36, 37]. We used the varying-coefficient multiplicative hazard model to determine the incident/dynamic area under the curve (AUC) for the accuracy of the (linear prediction) of the model score [38, 39]. We checked for outliers using the DFBETA test (S6 Fig) and plotted partial effect plots (S7–S9 Figs).

We assigned study numbers to enrolled participants. We also used participants’ laboratory numbers to access information from the laboratory information system (LIS) model Build 981 (Disa*Lab, Cape Town, South Africa). This process reduced missing data to 7%. Further, we aligned the follow-up dates with their planned review dates to reduce missing data. We imputed the missing values using multiple imputations by Chained Monte Carlo Equations (MICE).

## Results

### Comparison of baseline clinical and laboratory characteristics of participants with and without nephrotoxicity after 3 months of TDF-based therapy

Out of a total of 205 participants, 45 (22%, 95% confidence interval CI 17, 28%) developed TDF-associated nephrotoxicity for 838 person-months, equivalent to a TDF-associated nephrotoxicity incidence rate of 263 cases per 1000 person-months. The first case of TDF-associated nephrotoxicity was observed 16 days following TDF initiation. There were no differences in proportions by sex, cigarette smoking, alcohol use, herb intake, viral suppression, viremia, baseline eGFR (estimated eGFR ≤ 60 mL/min/1.73m^3^), granulated eGFR≥ 60 (S1 Table) or baseline serum creatinine ≥ 120 μmol/L between those who did and did not develop nephrotoxicity “Table 1”. Further, there were no statistically significant differences in the median laboratory values of age, sex-stratified BMI, mean arterial pressure (MAP), duration of follow up, viral load, CD4+ count, serum creatinine, fasting blood glucose (FBG), eGFR, serum cholesterol, urine creatinine, urine albumin-to-creatinine ratio, and urine phosphate-to-creatinine ratio between participants who did and did not develop nephrotoxicity “Table 1”.

**Table 1:** Comparison of baseline clinical and laboratory characteristics stratified by TDF-associated nephrotoxicity.

Variable	No Nephrotoxicity (N = 158, 77.8%)	Nephrotoxicity (N = 45, 22.2%)	*P*
	Clinical, frequency (%)		
Sex [Female]*	89 (56)	31 (69)	0.1*
Smoker	10 (8)	3 (9)	0.5^E^
Alcohol drinkers	52 (40)	11 (32)	0.6*
Herb user	25 (19)	8 (24)	0.4^E^
Viral Suppression	25 (16)	7 (16)	1.0^E^
Viremia	128 (81)	37 (82)	1.0^E^
Baseline eGFR< 60	4 (3)	2 (4)	0.6^E^
Baseline eGFR> 60	154 (97%)	43 (96%)	0.6^E^
Baseline SCr< 120	5 (3)	3 (7)	0.4^E^
Baseline SCr> 120	153 (97)	42 (93)	0.4^E^
	Median (IQR)		
Age [years]	36 (34, 37)	37 (32, 42)	0.4
Females BMI [kg/m^2^]	24 (21, 27)	24 (19, 27)	0.3
Males BMI [kg/m^2^]	22 (20, 25)	22 (18, 25)	0.5
MAP [mmHg]	98 (94, 102)	99 (93, 105)	0.5
Follow-up post-TDF [months]	3.5 (2.6, 4)	3.1 (2.3, 4)	0.2
	Laboratory, median (IQR)		
Viral load [copies/mL]	62163 (6939, 262543)	64857 (15354, 191603)	0.9
CD4+ [cells/L]	230 (135, 454)	304 (166, 441)	0.6
Serum creatinine [μmol/L]	65 (53, 77)	56 (43, 77)	0.1
eGFR [mL/min/1.73m^3^]	132 (108, 143)	140 (105, 153)	0.2
Cholesterol [mmol/L]	4.2 (3.3, 5)	3.7 (2.7, 4.6)	0.1
FBG [mmol/L]	4.5 (4.2, 4.8)	4.2 (3.9, 4.5)	0.3
Urine Creatinine [mmol/L]	9.7 (6.1, 15.4)	8.7 (6.6, 13.5)	0.9
albumin-to-creatinine [mg/g]	103 (50, 263)	153 (61, 351)	0.6
Phosp-to-creatinine [mol/mol]	1.7 (1.4, 2)	2.1 (1.3, 3)	0.2

SCr, serum creatinine; MAP, Mean Arterial Pressure; eGFR, Estimated Glomerular Filtration Rate.

*p values were calculated using the Chi-square test used

^E^*p* values were calculated using the Fisher’s exact test; *p* values were calculated using the Wilcoxon signed-rank test.

### Change in clinical and laboratory characteristics following three months of TDF-based therapy

After 3 months of TDF-based therapy, there was a statistically significant reduction in kidney function compared to baseline evidenced by an increase in serum creatinine by a mean of 29 μmol/L (95%CI 11, 47), and a mean reduction in eGFR of 15 mL/min/1.73m^2^ (95%CI 9, 20). There was an increase in the proportion of participants with serum creatinine>120 μmol/L from 8 (4%) to 16 (8%) at baseline and 3-months, respectively. There was an increase in CD4+ count by 86 cells/mm^3^ (95%CI 40, 132). However, there was no significant change in electrolytes, cholesterol, blood urea nitrogen (BUN), FBG, or urinary measures (creatinine, phosphate, and albumin-to-creatinine ratio). Information about change in viral load at 3-month was not available as standard practice recommends viral load assessment after every six months of therapy “Table 2”.

**Table 2:** Change in clinical and laboratory characteristics following 3-months of TDF-based therapy.

Variable	Baseline (N = 205)	3-Month (N = 203)	Diff. (95%CI)	*P-Value*
	Median (IQR)			
CD4+ [cells/mm^3^]	231 (138, 454)	335 (237, 433)	86 (40, 132)	<0.01
Serum creatinine [μmol/L]	65 (49, 77)	72 (56, 94)	29 (11, 47)	<0.01
eGFR [mL/min/1.73m^2^]	132 (108, 145)	120 (89, 140)	-15 (-9, -20)	<0.01
Serum Na^+^ [mmol/L]	134 (131, 136)	133 (130, 136)	1 (-5.3, 7.3)	0.5
Serum K^+^ [mmol/L]	4.2 (4.1, 4.4)	4 (3.8, 4.2)	0.2 (-0.1, .6)	0.4
Serum Cl^-^ [mmol/L]	101 (99, 102)	100 (98, 102)	-0.1 (-2.8, 2.7)	0.4
Serum PO_4_^2-^ [mmol/L]	0.93 (0.87, 1)	0.9 (0.79, 1)	-0.02 (-.13, .08)	0.7
BUN [mmol/L]	3.23 (2.1, 5.6)	3.4 (2.4, 6.8)	0.3 (-0.2, 0.8)	0.3
Urine Creatinine [mmol/L]	9.7 (7.8, 11.6)	8.5 (5.9, 11.2)	-1.2 (-10.8, 8.4)	0.9
Urine PO_4_^2-^ [mmol/L]	15.4 (6.5, 26.1)	14.2 (7.2, 21.3)	4.8 (-7.2, 17)	0.4
uACR [mg/g]	113 (52, 281)	118 (41, 222)	45 (-107, 234)	0.3
Frequency (Percent)				
Proteinuria > 1000 mg/g	18 (8.8%)	21 (10.3%)		0.2*
eGFR < 60ml/min/1.73m^3^	6 (3%)	17 (8.4%)		0.1*
Serum creatinine > 120 μmol/L	8 (4%)	16 (8%)		<0.01*

BUN, Blood Urea Nitrogen; uACR, Urine Albumin-to-Creatinine ratio; IQR, interquatile range.

* *p* values were calculated using the Fischer’s exact test; *p* values were calculated using the Wilcoxon’s signed-rank test for all continuous variables.

### Cox regression model for predicting nephrotoxicity at three months of TDF-based therapy

Every 10 μmol/L difference in baseline serum creatinine resulted in a 7% (95% CI 3, 12%) increase in the relative risk of TDF-associated nephrotoxicity. Every 10 mL/min/1.73m^3^ difference in baseline eGFR was associated with an 11% (95% CI 4, 20%) increase in the relative risk of TDF-associated nephrotoxicity. Being female was associated with 2.61 (95%CI 1.30, 5.24)-fold increased relative risk of developing TDF-associated compared to being male “Table 3”.

**Table 3:** Cox regression model for hazard ratio of TDF-associated nephrotoxicity.

Variable	Hazard Ratio	P-Value	[95% CI]
Creatinine _10pt	1.07	0.001	1.03, 1.12
eGFR_10pt	1.11	0.006	1.04, 1.20
BMI	0.96	0.260	0.91, 1.05
CD4_100pt	0.99	0.885	0.91, 1.08
MAP	1.01	0.475	1.00, 1.03
age	1.00	0.133	0.99, 1.05
*ln*uacr1	1.07	0.430	0.91, 1.25
*ln*vl1	1.00	0.988	0.93, 1.08
female	2.61	0.007	1.30, 5.24

Creatinine_10pt, Baseline serum creatinine/10; eGFR_10pt, Baseline eGFR/10; BMI, Baseline Body Mass Index; MAP, Baseline Mean Arterial Pressure; CD4_100pt, baseline CD4 count/100; *ln*uacr1, log transformed baseline urine albumin-to-creatinine ratio; *ln*vl1, Log transformed baseline viral load; age, age in months.

### Model diagnostics and performance

The log-log plot showed no violation of the proportional hazards assumption (S3 and S4 Figs). The Schoenfeld residuals showed no evidence of deviation from the assumption of proportional hazards p = 0.43. The model fit and discrimination by Harrell’s Concordance was 0.67, with Somers’ D of 0.34; the model heuristic shrinkage estimator gave an optimism of -0.04 after 400 bootstrap internal validations. The area under the time-dependent receiver operator curve (ROC) for the linear prediction of the model at three months of TDF-based therapy was 0.65 (95%CI 0.58, 0.68), with median sensitivity of 0.69 (IQR 0.43, 0.90), specificity of 0.52 (IQR 0.27, 0.77), median positive predictive value (PPV) of 0.59 (IQR 0.55, 0.62) and median negative predictive value (NPV) of 0.60 (IQR 0.56 0.69).

## Discussion

In this study, we derived a prognostic model for TDF-associated nephrotoxicity defined by the Acute Kidney Diseases and Disorder criteria [33, 34] at 3-months of TDF-based therapy among ART-naïve adults living with HIV and attending the largest HIV referral hospital in Zambia. Currently, clinicians are recommended to switch from TDF-based to abacavir-based ART regimens in patients who develop nephrotoxicity [40]. However, we demonstrated that predicting TDF-associated nephrotoxicity through a well derived and validated predictive model may help clinicians estimate and discriminate patients at risk of developing the complication before initiating therapy.

Clinical guidelines in Zambia recommend selecting patients with eGFR greater than 60 mL/min/1.73m^3^ and serum creatinine< 120 μmol/L to initiate TDF-based therapy [30]. However, this study showed that though patients initiate therapy according to this recommendation approximately 22% of them end up with iatrogenic acute kidney disease in the first three months. A comparison of baseline clinical and laboratory characteristics of the patients that suffered nephrotoxicity and those that showed no statistically significant difference suggests that these patients are indistinguishable at baseline when routine clinical and laboratory assessments are used. For instance, using the recommended (eGFR> 60 mL/min/1.73m^3^ and serum creatinine< 120 μmol/L), only six participants (3%) could be considered to have started TDF therapy with high risk for TDF-associated nephrotoxicity. Even limiting to those with baseline eGFR >60, there was still a substantial number that develops evidence of kidney dysfunction after initiating therapy. Further, out of the six participants who initiated TDF with high risk according to the clinical guidelines, only two developed nephrotoxicity at 3 months, which meant only 33% sensitivity to nephrotoxicity. These findings showed that the recommendation was a blunt tool that needed refinement to effectively discriminate patients at risk of TDF-associated nephrotoxicity before initiating therapy; however, a formal adequately powered study to compare the current recommendation to our predictive model is needed to prove this hypothesis.

We followed patients with preserved kidney function and no known history of diabetes or underlying comorbidities that were initiating TDF-based ART from December 2018 to November 2019 for approximately three months from the day they initiated therapy. Consistent with other studies [41, 42], we found a female preponderance in our study probably because females tend to be more willing to participate in studies than males. Compared to baseline, there was a 29 μmol/L increase in serum creatinine concentration observed at 3-months following therapy initiation, consistent with findings that TDF therapy leads to nephrotoxicity and reduced renal function in the first three to six months [27, 43]. Additionally, compared to baseline, there was a 15 mL/min/1.73m^3^ reduction in eGFR observed at 3-months of therapy, consistent with findings from Japan that suggest a rapid decline in eGFR with TDF exposure [26]. There was also an increase in CD4+ count by 86 cells/L observed at three months of TDF therapy, consistent with the CD4+ immune cell improvement often seen in TDF-based therapy [44, 45].

The scientific basis for building a predictive model for TDF-associated nephrotoxicity suggest that the current standard biomarker for detecting nephrotoxicity or renal disorders (serum creatinine and eGFR) were not good enough because they seem to deflect late, often after the renal damage has taken place; therefore, predicting impending TDF-associated nephrotoxicity and injury might provide a prevention strategy [28, 30, 46]. With 22% of participants developing TDF-associated nephrotoxicity, the incidence concurred with a Japanese study [26], but was higher and not consistent with some African studies [47–49], probably because of the different criteria used to define nephrotoxicity and different censoring timings. The higher incidence in our study could also be ascribed to the fact that we censored the outcome at approximately three months of initiating therapy, a period that is reported to be the epitome for TDF-associated nephrotoxicity [28, 29]. However, the difference in the race of study populations with ours being wholly Africans who may be genetically predisposed to HIV-associated nephropathy (HIVAN) and renal diseases could further explain the inconsistent incidence rate reported [20, 23, 50–52].

The factors that significantly increased the risk of developing TDF-associated nephrotoxicity in the full model were an increase in baseline serum creatinine; a decrease in baseline eGFR, and female sex. These findings suggest an association between the baseline characteristics and baseline renal biomarkers of the participant and developing TDF-associated nephrotoxicity after three months of TDF-based therapy. The hazard ratio (relative risk) for TDF-associated nephrotoxicity for every 10 μmol/L difference in baseline serum creatinine increased by 7%; since the relative risk is the probability of an event occurring versus the probability of no event, the probability of nephrotoxicity for every 10 μmol/L difference in baseline serum creatinine was 52% (95% CI 51, 53%). It can be argued that serum creatinine was part of the CKD-EPI formula for deriving eGFR and therefore related to renal function, however, since the participants were treated with TDF for three months which affects renal function, discovering that a change of 7% in the risk of TDF-associated nephrotoxicity was due to baseline creatinine was a relevant finding [13, 53]. Further, every 10 mL/min/1.73m^3^ difference in baseline eGFR was associated with an increase in the relative risk of TDF-associated nephrotoxicity of 11% equivalent to a probability of nephrotoxicity of 53% (95% CI 51, 55%). Usually, a linear relationship would exist between two paired observations, which could explain the association between baseline and three-month eGFR [54, 55]; however, similar findings were reported in other studies [25, 56, 57]. Finally, females had a higher relative risk of TDF-associated nephrotoxicity than males (HR 2.61), equivalent to a probability of TDF-associated nephrotoxicity of 72% (95% CI 57%, 84%) suggesting that female sex increased the chances of TDF-associated nephrotoxicity, consistent with findings from South Africa and the USA [51, 58]. Nevertheless, close monitoring of patients of either sex is cardinal to mitigate the risk of kidney complications in patients initiating TDF-based therapy. We recommend further research to investigate the observed disparity.

We prospectively derived a prognostic model at the largest HIV referral clinic in Zambia and used robust internal validation with 400 random datasets using Efron’s bootstrap technique, as recommended in regression modelling strategies [54, 55]. This model provides for more effective, reliable and inexpensive methods for predicting renal injury before initiating TDF-based ART. However, further studies to externally validate this model would be desirable. The model was also derived from routinely done clinical and laboratory tests, and despite a modest performance it can be utilized in any kind of setting as well as be easily integrated into the electronic health record to be used by physicians before initiation of therapy without the addition of cost or treatment complexity.

The limitations of our study include the lack of a cohort for external validation of the model to test the performance and utility of the developed prognostic model in the real world and a lack of studies based on implementation science implementing the model into clinical practice. However, a study to externally validate the model and implement it into clinical practice is yet to begin. However, since the model was internally validated using bootstrap validation, it will be made available online for clinicians. Further, the Cox regression model could only predict nephrotoxicity if TDF-associated nephrotoxicity was defined according to the AKD criterion, as we did. Therefore, a study estimating the actual trend of the 3-month eGFR might supplement for those that decide not to employ the AKD criterion. Further, the model predicted nephrotoxicity only up to the first three months of TDF use and did not inform whether the nephrotoxicity proceeded to chronic kidney disease downstream or not. This is an area for future research; however, finding TDF-associated nephrotoxicity itself is important because many studies have demonstrated that nephrotoxicity can lead to renal and non-renal complications [29, 59, 60]. Finally, this model is not validated for use among children and needs validation for that population.

Our findings introduced a prognostic model for estimating the risk for TDF-associated nephrotoxicity in Zambia and if externally validated and performance-tested, they could inform policy to individualize treatment. Further, since TDF is the backbone of the first-line ART regimen in Zambia and other African countries, we recommend cohort studies to external validate the prognostic model and test the performance against prevailing algorithms in real-world clinical practice before widespread adoption.

In conclusion, we found that the baseline clinical demographics and kidney biomarkers of persons at the time of initiating therapy can predict the risk of TDF-associated nephrotoxicity after three months of TDF-based therapy. The baseline serum creatinine, baseline eGFR and female sex predicted the risk for TDF-associated nephrotoxicity following three months of therapy. This study provides a benchmark predictive model and once externally validated and implemented using implementation science methods would help physicians estimate the risk of TDF-associated nephrotoxicity prior to initiating patients on treatment in Zambia and similar African settings.

## Supporting information

S1 Fig Participant exclusion flowchart. Selection of participants included in the study.
(TIF)

S2 Fig Hierarchical variable clustering for Cox model. Identified highly correlated predictors and selected one to include in the model.
(TIF)

S3 Fig Predictive potential for nephrotoxicity. Identified predictors with high predictive potential to be included in the model.
(TIF)

S4 Fig Unrestricted Kaplan-Meier nephrotoxicity survival curve by sex. Graphically displayed no diviation from the proportional hazards; the predicted and observed survival curves in Kaplan-Meier were close to each other.
(TIF)

S5 Fig Log-log plots of nephrotoxicity-free survival curve by sex. Fairly parallel plot that did not cross suggesting there was no violation of the proportional hazard’s assumptions.
(TIF)

S6 Fig DFBETA plots for outliers in creatinine, eGFR, age and sex. No outliers or highly influential creatinine observations as the fitted line was symmetrical around zero with most points clouded around the horizontal line near zero.
(TIF)

S7 Fig The plot of log relative hazard against age by sex. Graphically showed that females had higher risk of nephrotoxicity than males at different ages.
(TIF)

S8 Fig The plot of log relative hazard against serum creatinine by sex. Graphically showed that females had higher risk of nephrotoxicity than males at different baseline serum creatinine concentrations.
(TIF)

S9 Fig The plot of log relative hazard against baseline eGFR by sex. Graphically showed that females had higher risk of nephrotoxicity than males at different baseline eGFR.
(TIF)

S1 Table Granulated baseline serum creatinine stratified by nephrotoxicity. Compared the proportions of patients with baseline eGFR greater than 60 mL/min/1.73m^3^ and granulated the eGFR in 10 mL/min/1.73m^3^ among cases and controls.
(TIF)

S2 Table Variance-covariance estimate correlation matrix. Demonstrated that there was no multicollinearity among predictors included in the model.
(TIF)
