# Supplementary figures and images for: Prognostic model for nephrotoxicity among HIV-positive Zambian adults receiving tenofovir disoproxil fumarate-based antiretroviral therapy
Source: PLoS One. 2021 Jul 12;16(7):e0252768. doi: 10.1371/journal.pone.0252768 (PMC8274919; doi:10.1371/journal.pone.0252768)

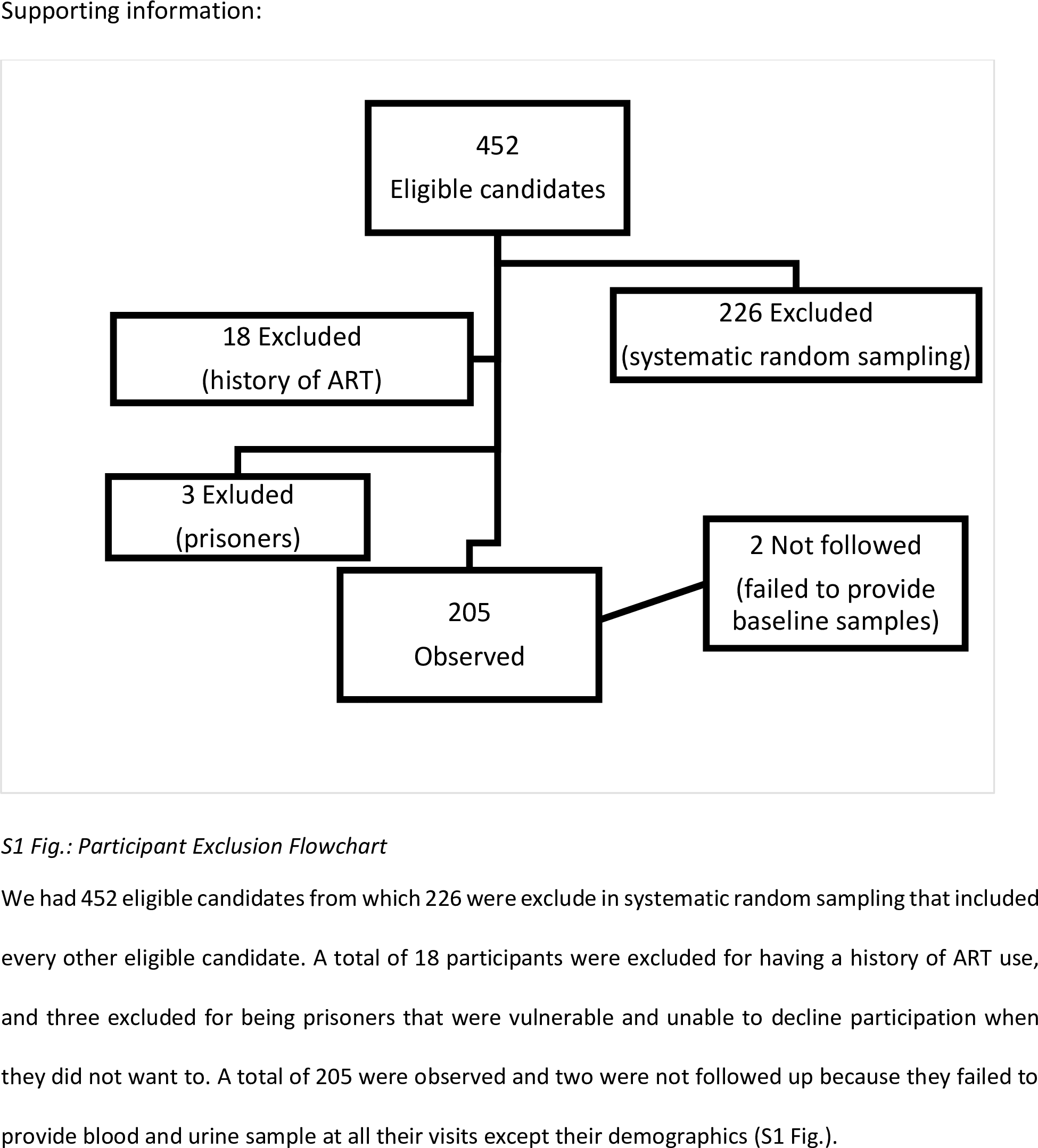

Supplement: S1 Fig — Selection of participants included in the study. (TIF) [file pone.0252768.s001.tif]

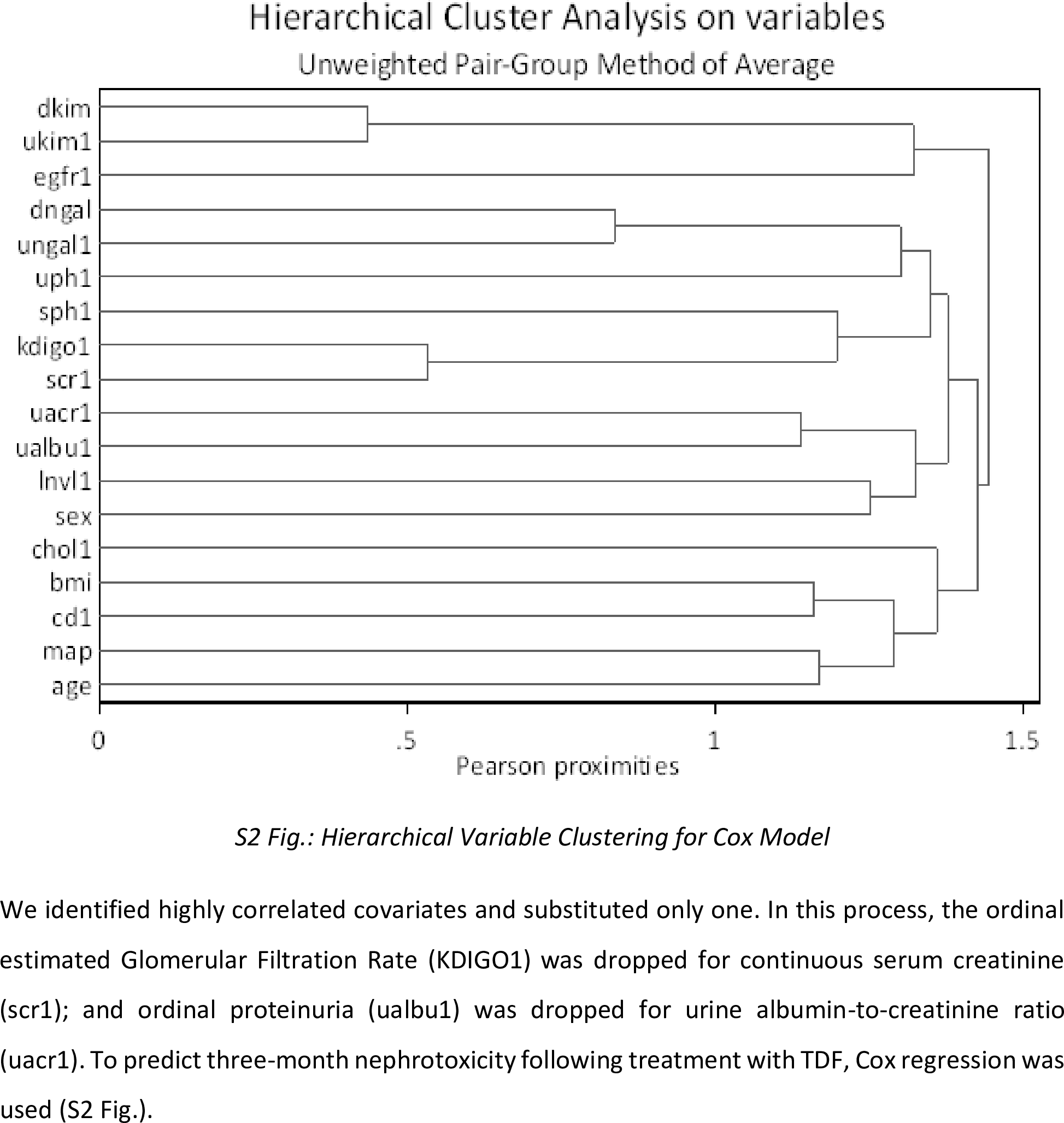

Supplement: S2 Fig — Identified highly correlated predictors and selected one to include in the model. (TIF) [file pone.0252768.s002.tif]

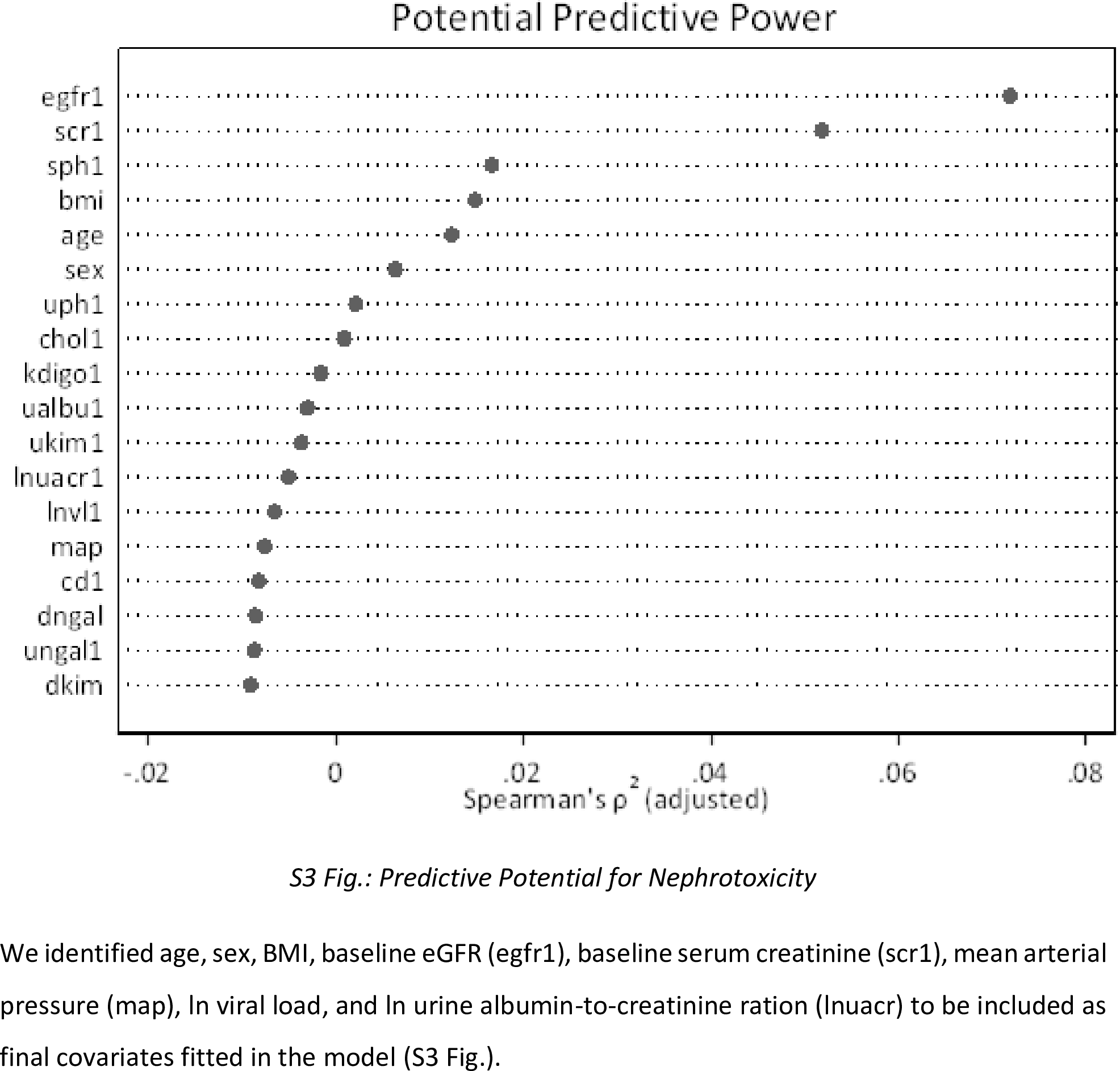

Supplement: S3 Fig — Identified predictors with high predictive potential to be included in the model. (TIF) [file pone.0252768.s003.tif]

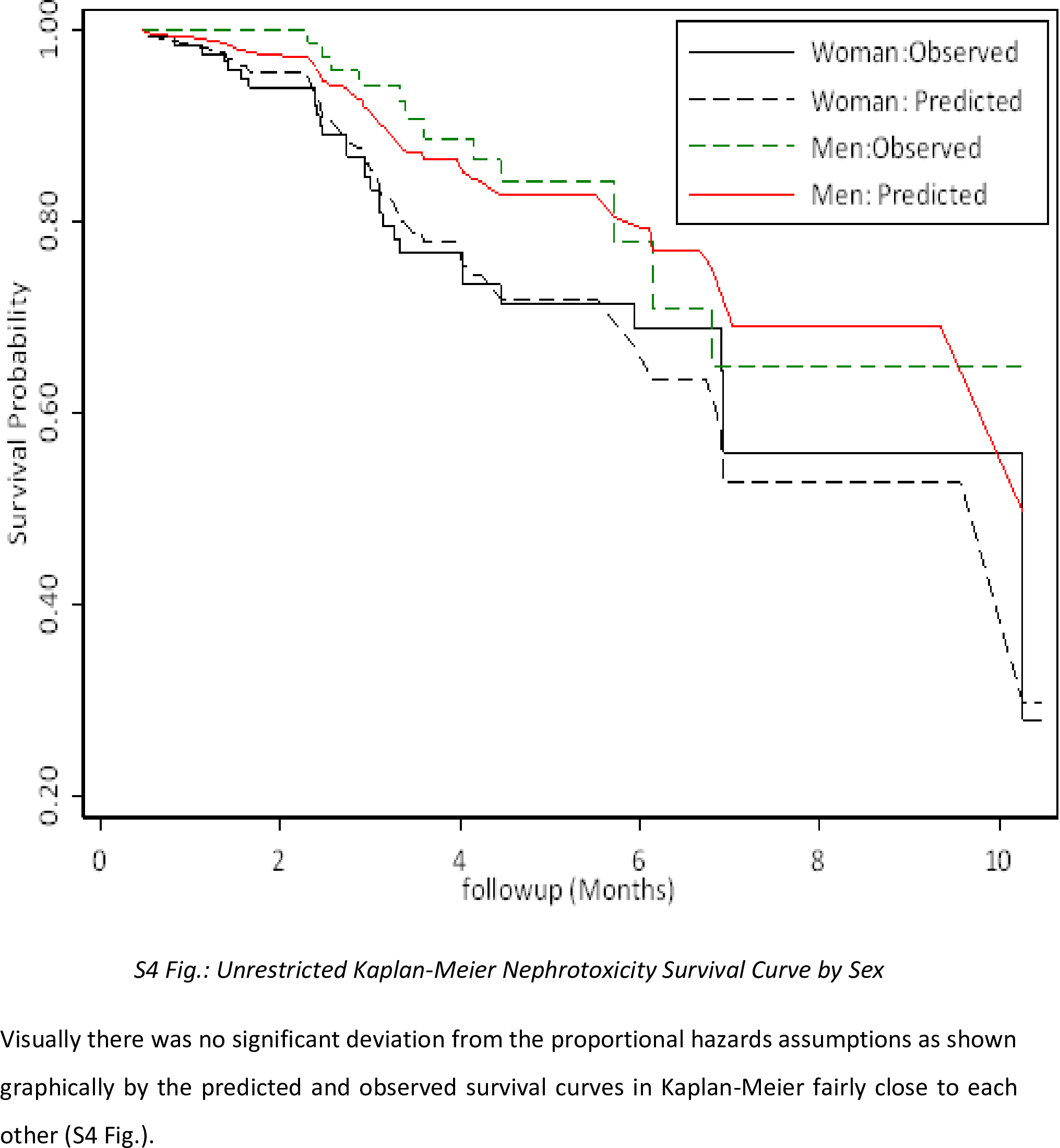

Supplement: S4 Fig — Graphically displayed no diviation from the proportional hazards; the predicted and observed survival curves in Kaplan-Meier were close to each other. (TIF) [file pone.0252768.s004.tif]

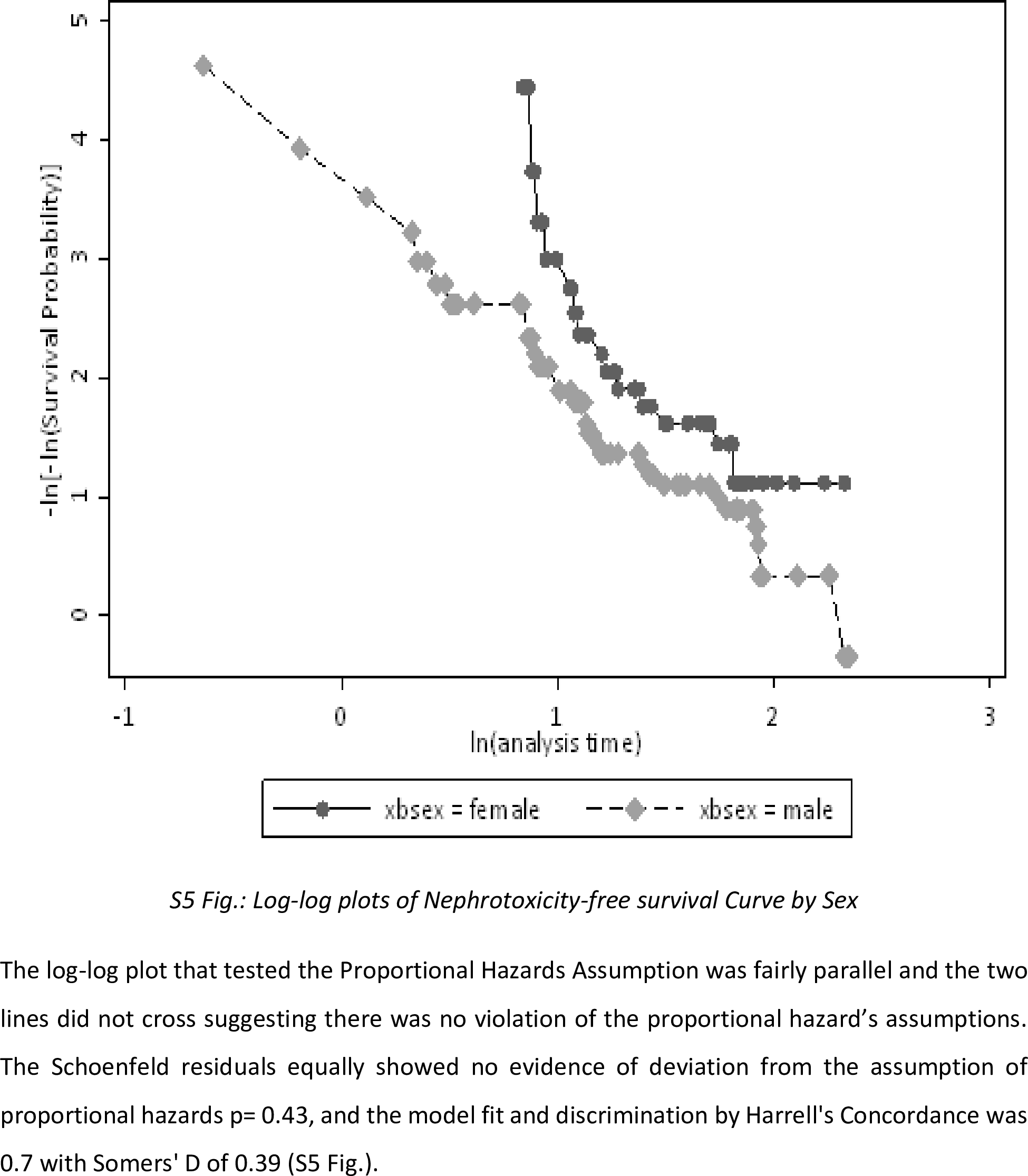

Supplement: S5 Fig — Fairly parallel plot that did not cross suggesting there was no violation of the proportional hazard’s assumptions. (TIF) [file pone.0252768.s005.tif]

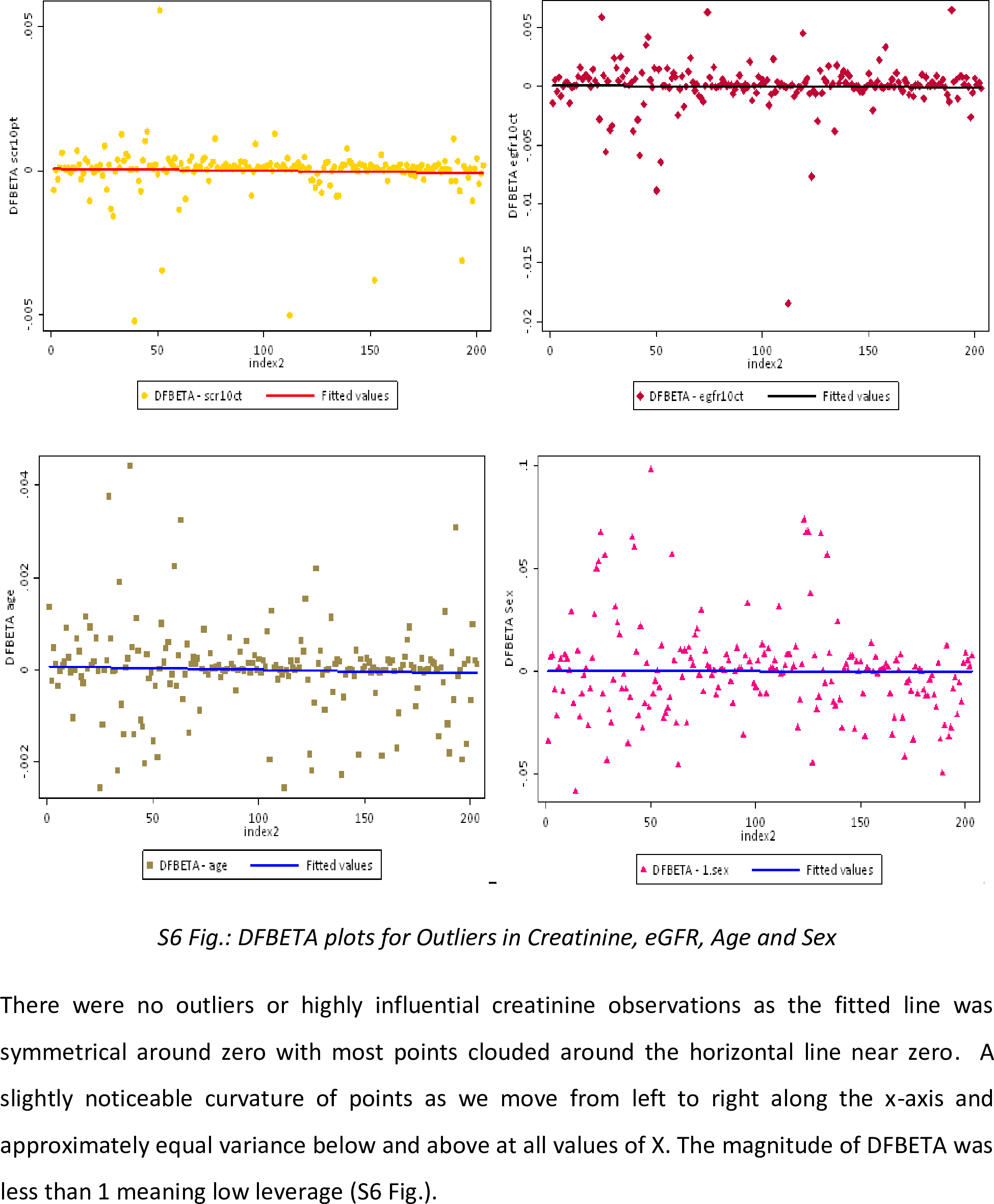

Supplement: S6 Fig — No outliers or highly influential creatinine observations as the fitted line was symmetrical around zero with most points clouded around the horizontal line near zero. (TIF) [file pone.0252768.s006.tif]

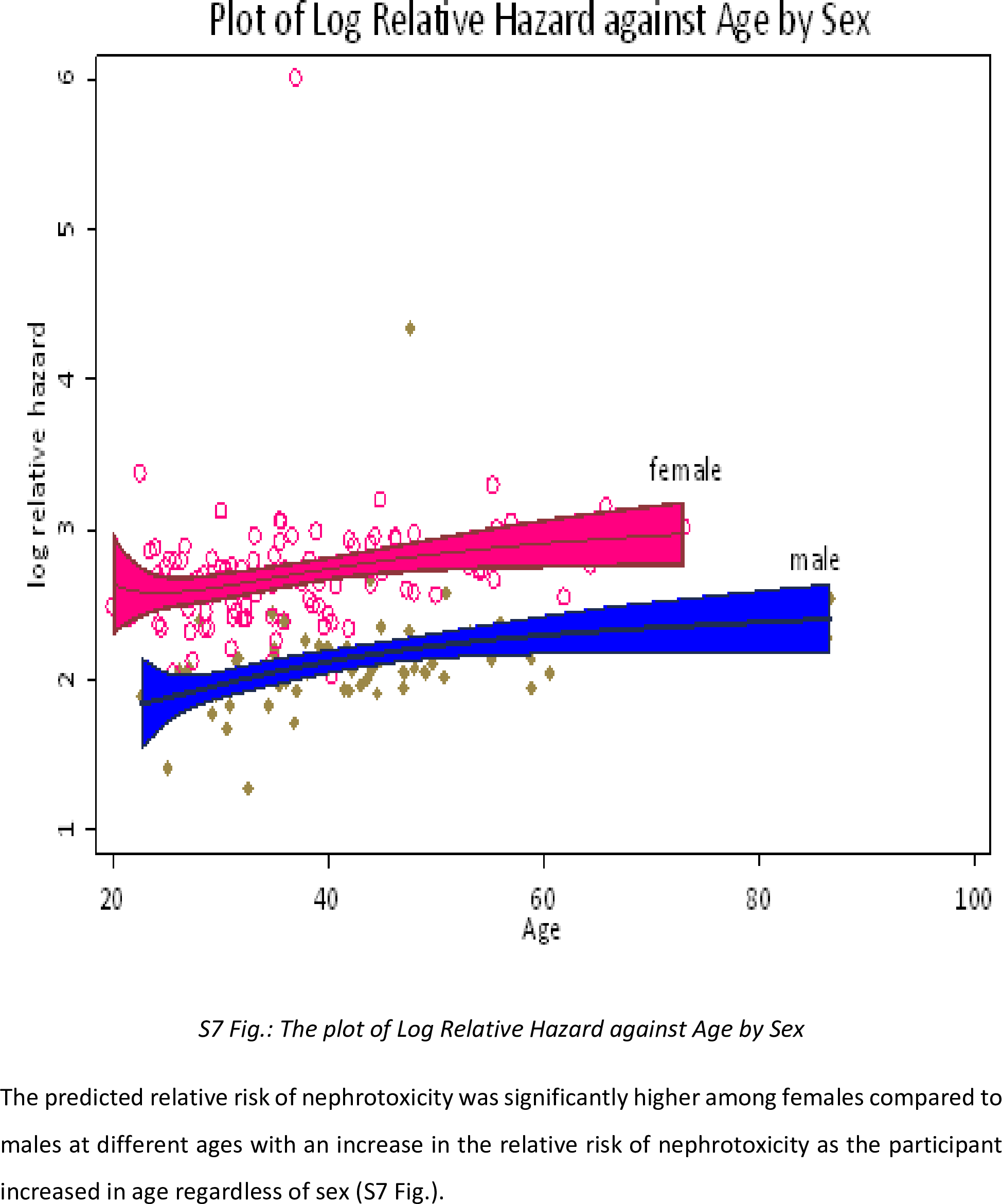

Supplement: S7 Fig — Graphically showed that females had higher risk of nephrotoxicity than males at different ages. (TIF) [file pone.0252768.s007.tif]

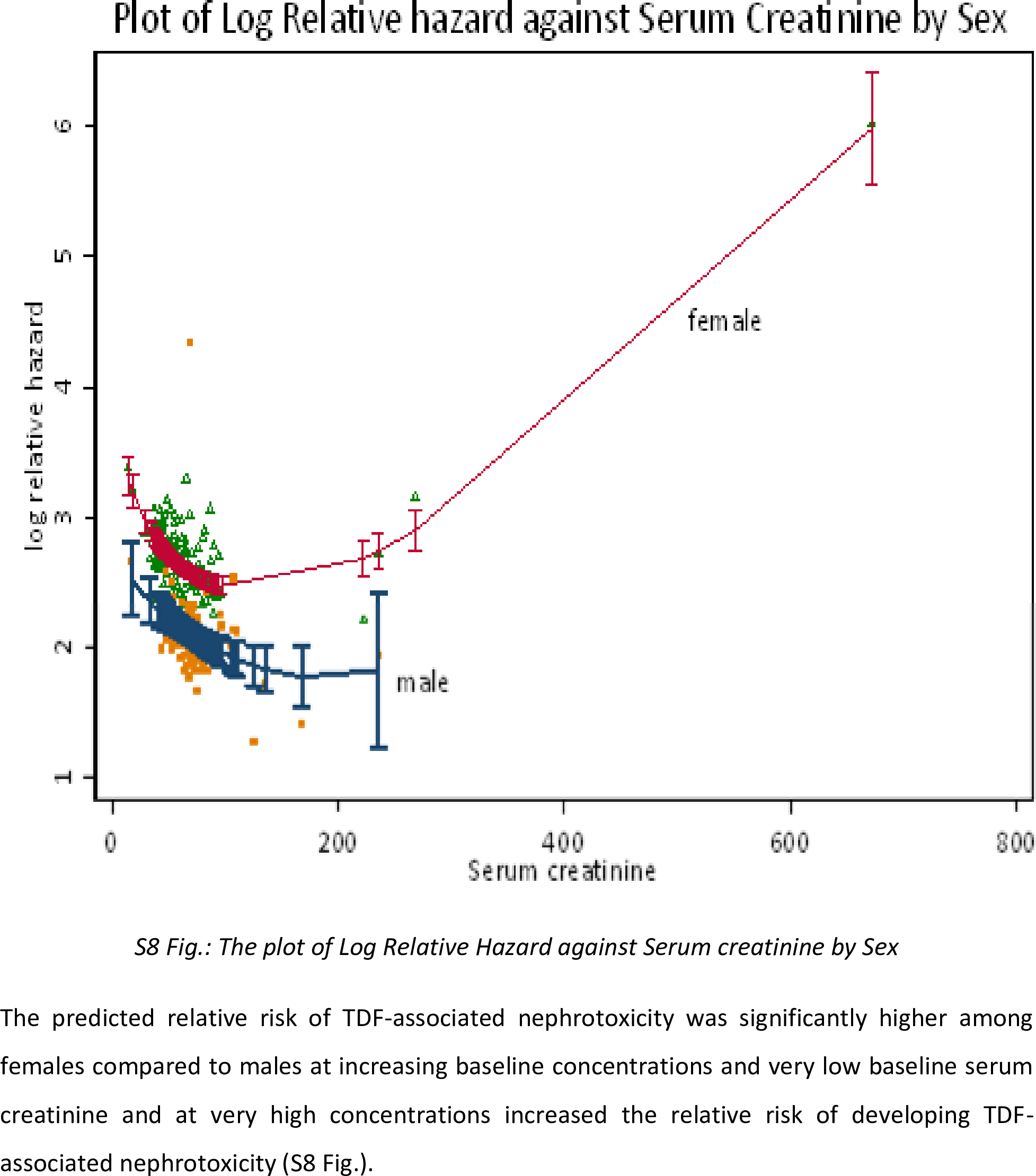

Supplement: S8 Fig — Graphically showed that females had higher risk of nephrotoxicity than males at different baseline serum creatinine concentrations. (TIF) [file pone.0252768.s008.tif]

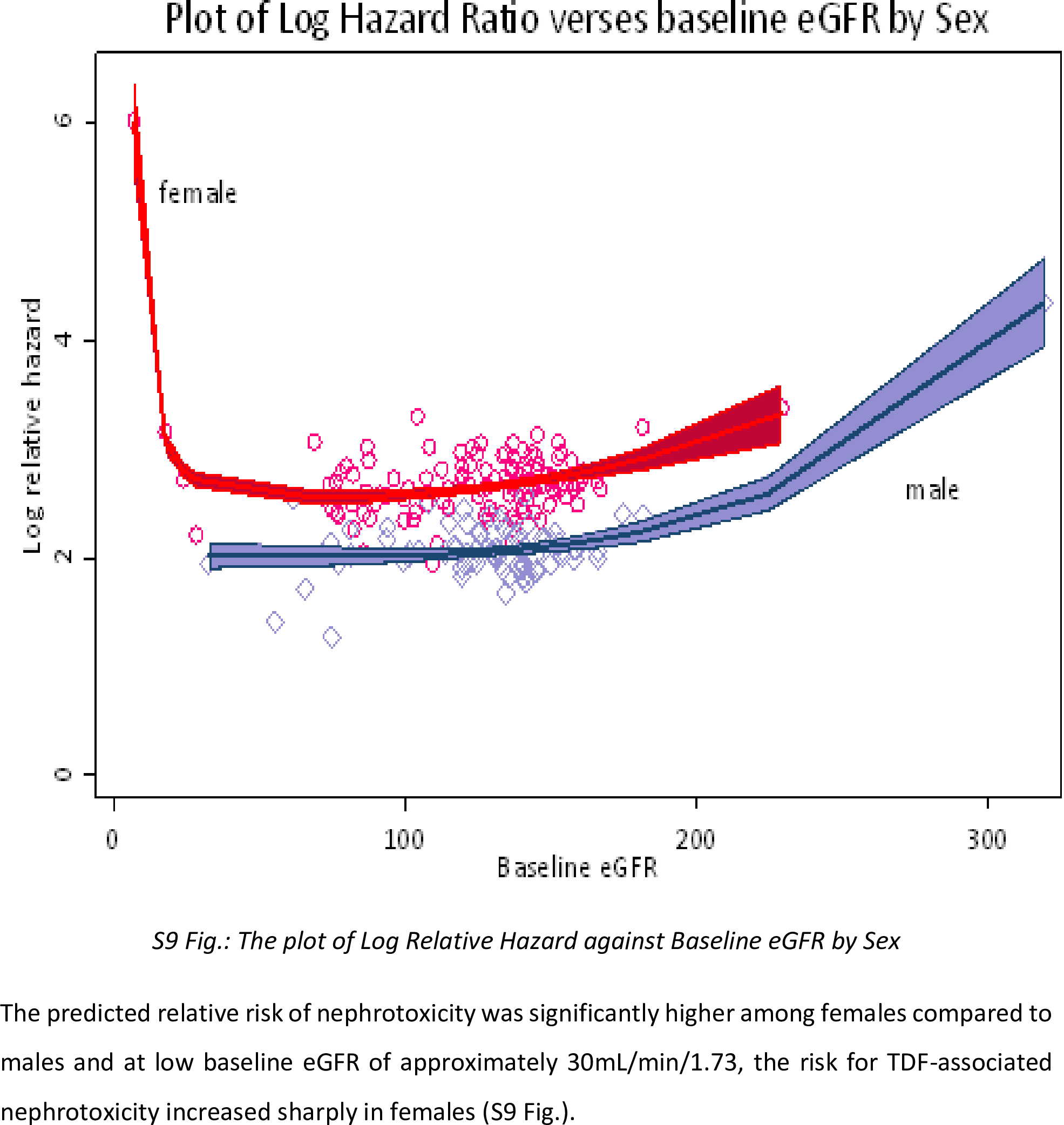

Supplement: S9 Fig — Graphically showed that females had higher risk of nephrotoxicity than males at different baseline eGFR. (TIF) [file pone.0252768.s009.tif]

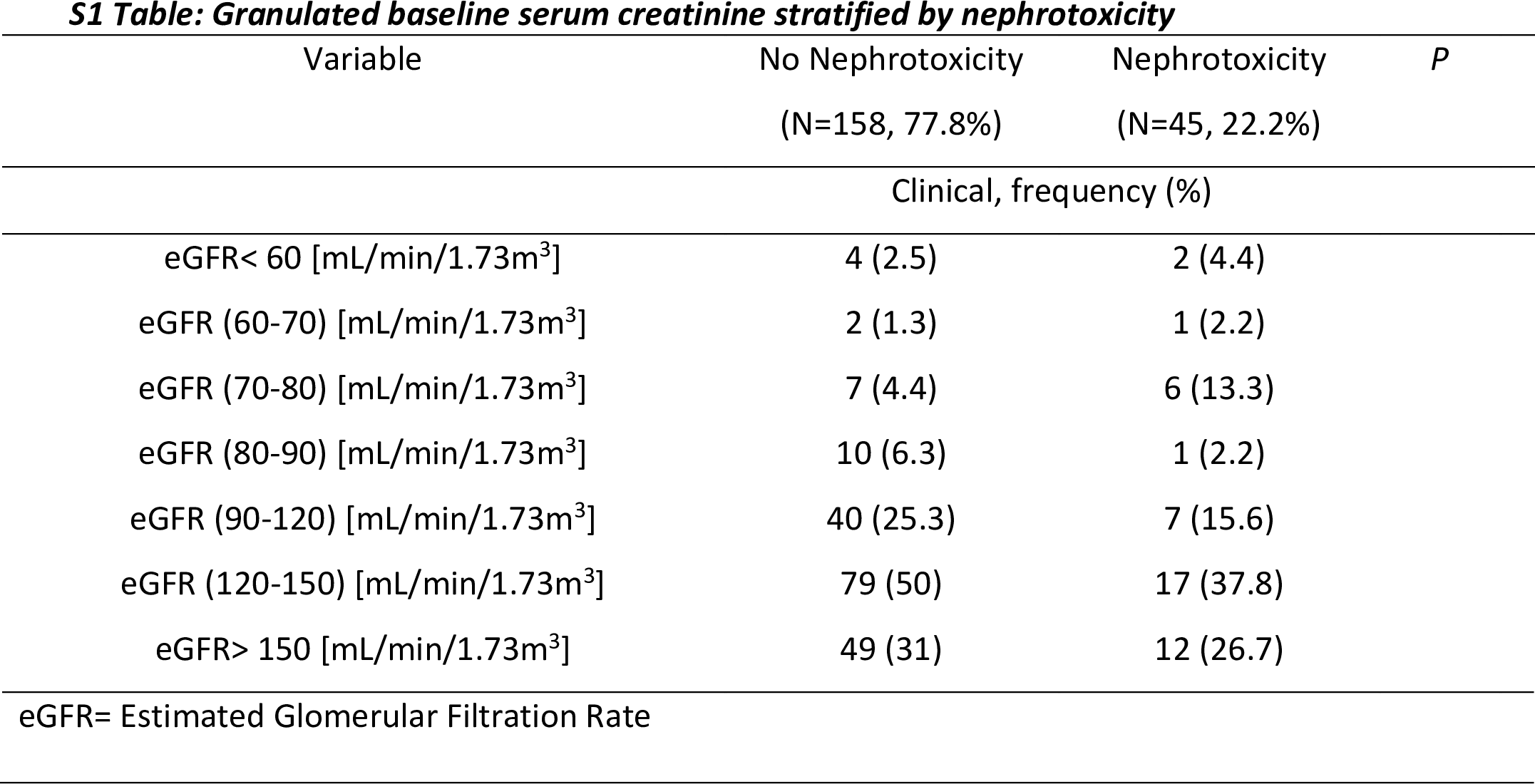

Supplement: S1 Table — Compared the proportions of patients with baseline eGFR greater than 60 mL/min/1.73m3 and granulated the eGFR in 10 mL/min/1.73m3 among cases and controls. (TIF) [file pone.0252768.s010.tif]

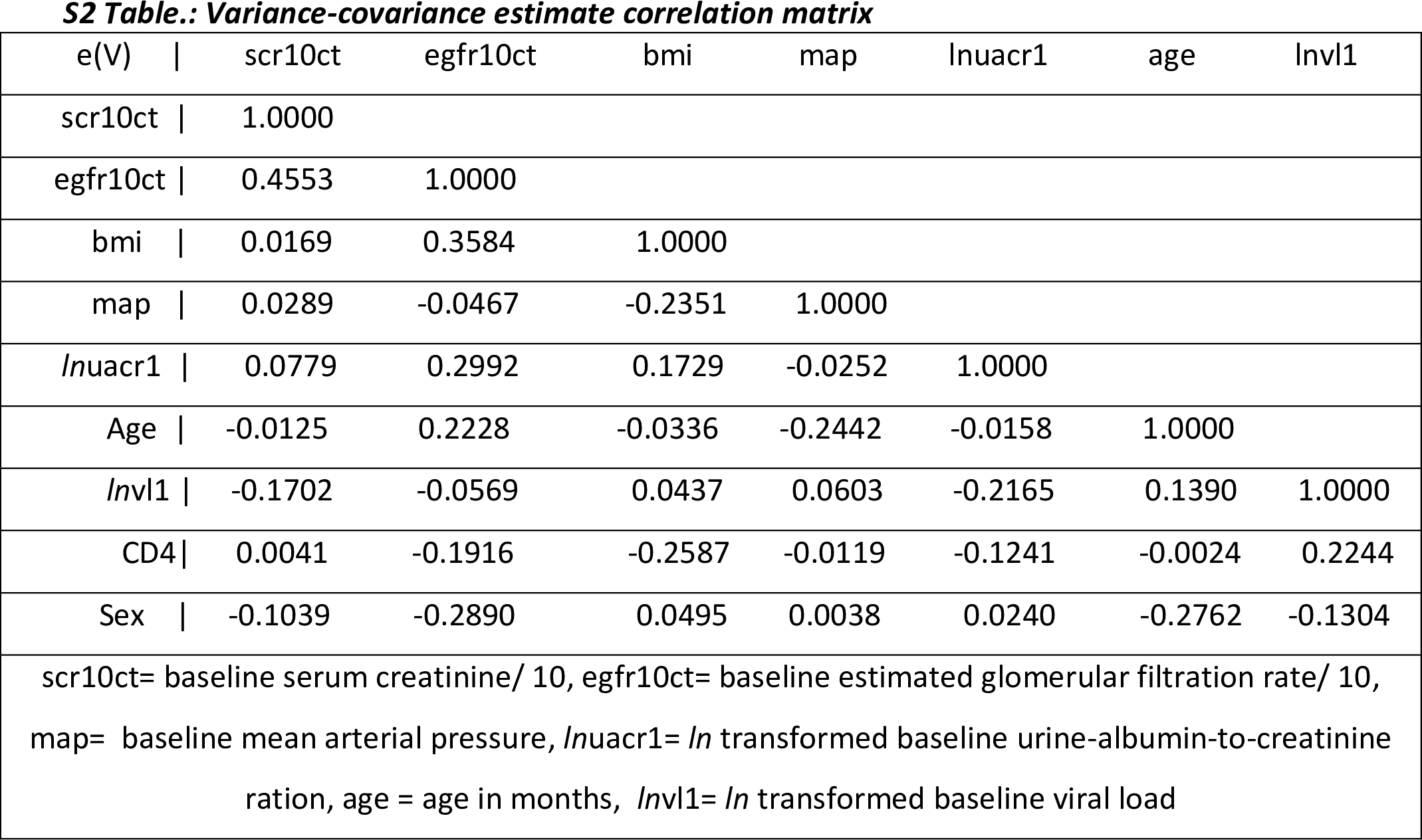

Supplement: S2 Table — Demonstrated that there was no multicollinearity among predictors included in the model. (TIF) [file pone.0252768.s011.tif]
